# Evaluation of Difference in Emergency Care Quality by Years of Physician Experience at the Emergency Department

**DOI:** 10.14789/jmj.JMJ24-0035-OA

**Published:** 2024-09-30

**Authors:** SHUNGO TOKUNAGA, AI IKEDA, DAISUKE USUDA, KENJI KAWAI, RIKI SAKURAI, SHIHO TSUGE, SHUN MATSUBARA, MAKOTO SUZUKI, RISA TANAKA, SHINTARO SHIMOZAWA, YUTA HOTCHI, IPPEI OSUGI, AKIHIKO KONDO, KENTARO MISHIMA, KEIKO MIZUNO, TAKAYUKI KOMATSU, HIROKI TAKAMI, JIRO OBA, TOMOHISA NOMURA, MANABU SUGITA

**Affiliations:** 1 Department of Emergency and Disaster Medicine, Juntendo University Graduate School of Medicine, Tokyo, Japan; 1 Department of Emergency and Disaster Medicine, Juntendo University Graduate School of Medicine, Tokyo, Japan; 2 Department of Emergency and Critical Care Medicine, Juntendo University Nerima Hospital, Tokyo, Japan; 2 Department of Emergency and Critical Care Medicine, Juntendo University Nerima Hospital, Tokyo, Japan; 3 Department of Public Health, Juntendo University Graduate School of Medicine, Tokyo, Japan; 3 Department of Public Health, Juntendo University Graduate School of Medicine, Tokyo, Japan

**Keywords:** emergency medicine, length of stay, emergency triage

## Abstract

**Objective:**

To evaluate the effect of emergency medicine training credentials and years of medical experience on various clinical parameters in emergency medicine practice.

**Methods:**

A cross-sectional study was conducted at Juntendo University Nerima Hospital between 1 April 2019 and 31 March 2020. All patients who were transported by ambulance, were examined by emergency physicians, and underwent computed tomography (CT) or magnetic resonance imaging (MRI) in the emergency department were included. For these cases, data on the attending physician's qualification status and experience (specialist, nonspecialist with 1-2 years of experience, or nonspecialist with 3-4 years of experience), clinical parameters, and imaging were collected. The primary outcome was the patient's total length of stay (LOS) in the emergency department.

**Results:**

A total of 3,784 patients were included in the study. Patients attended by nonspecialists with 1-2 years of experience had a significantly longer time from arrival to assessment and LOS, especially in mild and severe cases and cases requiring head and abdominal CT imaging.

**Conclusion:**

Our findings suggest that for physicians with minimal work experience, mentorship and effective training using triage flow and medical examination protocols may help to reduce LOS in the emergency department.

## Introduction

As a referral center for emergency medicine in Nerima City, Juntendo University Nerima Hospital plays a vital role in community medicine by treating patients transported by ambulance or transferred from other regional medical institutions and managing patients with severe injuries and without access to local critical care centers. Additionally, the Department of Emergency Medicine is responsible for the management of inpatients who do not require cardiac or brain surgery or cardiac catheterization.

The Department of Emergency Medicine is required to make prompt and accurate diagnoses to provide tailored management of a wide variety of patients with trauma and various diseases^1, 2)^. In the clinical setting, an accurate patient history and thorough physical examination are essential for accurate diagnosis. In cases where arriving at a diagnosis is challenging, computed tomography (CT) and magnetic resonance imaging (MRI) often play a critical role in diagnosis^3-5)^. After arrival, it takes time to resuscitation of patient condition and do type of examination^6-8)^. Under such circumstances, extended hospital stays adversely affect prognosis. More effective strategies for circumventing such situations are therefore needed^9-11)^. At our hospital, two systems have been established to support emergency physician during times of heavy patient loads in the emergency department. First, a support emergency physician is assigned to each emergency physician in the department. Second, physicians from all departments are on call for consultation during all shifts.

We examined the duration between patient assessment and point of change in this study, where point of change is defined as a change in status, such as discharge, hospitalization, death, or transfer to another hospital. We sought to evaluate the effect of physicians' credentials and experience on length of time between patients' arrival and assessment, length of time between their assessment and the point of change, and their total length of stay (LOS) in the emergency department.

## Materials and Methods

**Figure 1:**
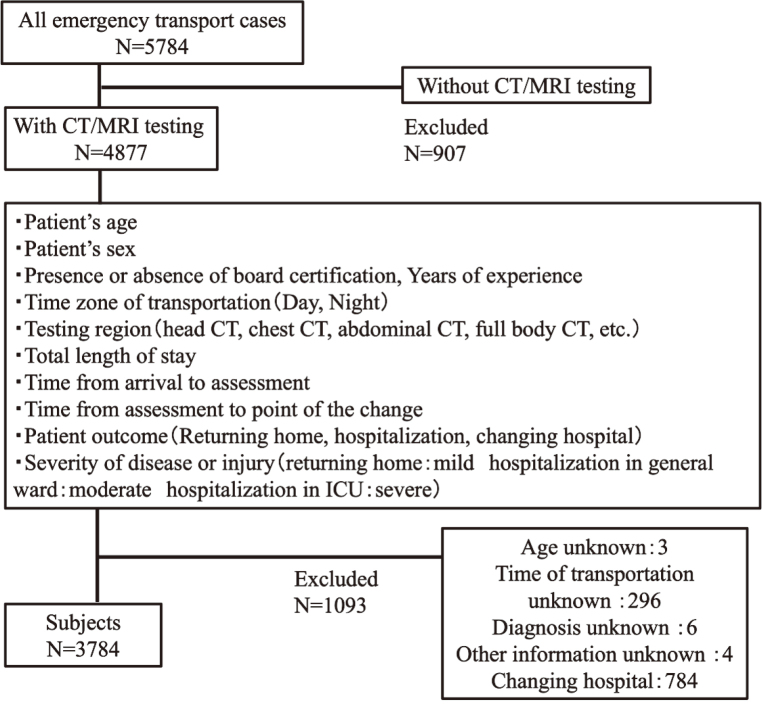
Case registration flowchart Patients admitted at the emergency department at Juntendo University Nerima Hospital between April 1, 2019 and March 31, 2020. And 2000 patients were excluded from participation in this study. CT, computed tomography; MRI, magnetic resonance Imaging

This cross-sectional study was conducted using data from patients admitted to the emergency department at Juntendo University Nerima Hospital between 1 April 2019 and 31 March 2020. During this period, 5,784 patients were transported by ambulance to the emergency department. Of these patients, 2,000 were excluded from participation in this study. The excluded patients included 907 who did not undergo CT or MRI, three whose age and sex were not recorded, 296 whose time of transportation was not recorded, six whose diagnosis was not recorded, four whose clinical information was inadequate, and 784 who were transferred to other hospitals. A detailed flowchart of the case registration process is given in Figure 1.

Assessment of the participants included CT or MRI (regardless of the body region imaged) ordered by physicians in the emergency and intensive care departments. Some information was gathered from the participants' electronic health records. The 3,784 cases of interest were classified into three groups based on the qualification and experience of the attending physician (board-certified specialist, non-board-certified specialist with 1-2 years of experience, and non-board-certified specialist with 3-4 years of experience). These three groups were compared with respect to the following parameters: patient's age, patient's sex, time of arrival (day: 8:00-17:00; night: 17:00-8:00 the following day; UTC +9), time from arrival to assessment, time from assessment to point of change, LOS in the emergency department, and type of examination (head CT, chest CT, abdominal CT, full-body CT, CT or MRI of other body regions). We also evaluated the severity of disease or injury (discharge, mild; hospitalization in general ward, moderate; and hospitalization in intensive care unit [ICU], severe). LOS was defined as the total length of stay from the time of arrival at the emergency department to the point of change.

We conducted analysis of variance and chi-square tests to compare group means and the proportions of demographic variables. We used analysis of covariance with Dunnet's multiple comparison test to assess differences in time from arrival to assessment, time from assessment to point of change, and LOS between patients managed by board-certified specialists and non-board-certified specialists with 1-2 or 3-4 years of experience. Natural logarithmic conversion was applied to these three measures to normalize the data distribution. In the multivariate models, the variables included were patient's age, patient's sex, severity of disease or injury, LOS, and body region imaged by CT. We further stratified LOS by the certification status of the attending specialist.

Statistical significance was set at *p* < 0.05. All statistical analyses were performed using SAS software version 9.4 (SAS Institute, Cary, NC, USA). This study was conducted with the approval of the Juntendo University Ethics Committee (Research Number: E22-0291) and in accordance with the principles of the Helsinki Declaration. The need for informed consent was waived because of the absence of invasive procedures and the use of anonymized data, which involved no to minimal risk for study participants.

## Results

The clinical characteristics of all 3,784 study participants are shown in Table 1. Significant differences in age, severity of disease or injury, time of examination, and body region imaged by CT were observed. In body regions imaged by CT, differences were observed in abdominal CT and full-body CT.

The results of the analysis of covariance, adjusted for patient age and sex with physician status and experience to validate the differences in outcomes (time from arrival to assessment, time from assessment to point of change, and LOS), are shown in Table 2. Patients attended by noncertified specialists with 1-2 years of experience had a significantly longer time from arrival to assessment and LOS than those attended by specialists with more experience. However, patients treated by noncertified specialists with 3-4 years of experience had a shorter LOS than those treated by certified specialists. There was no significant difference in time from assessment to point of change among the three groups.

The results of further stratification by severity of disease or injury are shown in Table 3. Among patients with severe disease or injuries, those attended by noncertified specialists with 1-2 years of experience had a significantly longer LOS and time from arrival to assessment. Among mild cases, those attended by noncertified specialists with 3-4 years of experience had a significantly shorter time from arrival to assessment, whereas noncertified specialists with 1-2 years of experience were associated with significantly longer outcomes for all durations considered. No differences were observed among cases of moderate severity in all groups.

The results of stratification by body region imaged by CT and physician status and experience are shown in Table 4. For patients examined via head CT, those treated by noncertified specialists with 1-2 years of experience had a significantly longer time from arrival to assessment and LOS, while those treated by noncertified specialists with 3-4 years of experience had a significantly shorter LOS only. Among cases with abdominal CT, those attended by noncertified specialists with 1-2 years of experience had a significantly longer time from arrival to assessment and LOS, while those attended by noncertified specialists with 3-4 years of experience had a shorter time from arrival to assessment.

**Table 1:** Basic patient characteristics

	Board certified specialists (N = 2188)	Non-board-certified specialists (N = 1596)		*p* for difference
		Non-board-certified specialists 1-2 years (N = 948)	Non-board-certified specialists 3-4 years (N = 648)	
Mean patient's age (years) ± SD	66 ± 22	68 ± 22	68 ± 21	0.02
Patient's sex Male (%)	1097 (50)	513 (54)	330 (51)	0.12
Time of transportation day (%)	916 (42)	466 (49)	280 (43)	< 0.001
Body region imaged				
Head CT (%)	1076 (49)	428 (45)	302 (47)	0.10
Chest CT (%)	208 (9)	83 (9)	67 (10)	0.57
Abdominal CT (%)	286 (13)	107 (11)	62 (10)	0.04
Full body CT (%)	314 (14)	170 (18)	103 (16)	0.04
Others (%)	304 (13)	160 (17)	114 (18)	0.02
Severity of disease or injury				
Mild (%)	1208 (55)	462 (39)	368 (57)	0.001
Moderate (%)	642 (29)	296 (31)	181 (28)	0.34
Severe (%)	338 (16)	190 (20)	99 (15)	0.004

Analysis of variance and chi-square tests were used to compare three groups means and proportions of demographic variables.

**Table 2:** Least square mean estimates and standard errors by years of physician experience

	Board certified specialists	Non-board-certified specialists (1-2 years)	Non-board-certified specialists (3-4 years)	*p* for difference (Board certified specialists vs. 1-2 years)	*p* for difference (Board certified specialists vs. 3-4 years)
Total, n	2188	948	648		
Time from arrival to assessment, min (SE)					
Crude	33.6 (1.02)	37.7 (1.03)	32.2 (1.03)	<0.001	0.39
Multivariate model**	35.0 (1.02)	38.9 (1.03)	33.1 (1.03)	<0.001	0.18
Time from assessment to point of change, min (SE)					
Crude	87.8 (1.02)	92.9 (1.02)	81.2 (1.03)	0.07	0.02
Multivariate model*	92.2 (1.01)	95.1 (1.02)	85.3 (1.03)	0.19	0.73
Length of stay, min (SE)					
Crude	136.7 (1.01)	147.6 (1.02)	129.0 (1.02)	<0.001	0.02
Multivariate model**	145.5 (1.01)	153.5 (1.02)	137.1 (1.02)	0.004	0.006

*Adjusted for patient's age, patient's sex, severity of disease *or injury*, length of stay, and body region imaged. **Adjusted for patient's age, patient's sex, severity of disease *or injury*, and body region imaged. Analysis of covariance with Dunnet's multiple comparison test was used to assess the differences in time from arrival to assessment, time from assessment to point of change, and LOS between patients managed by board certified specialists and non-board certified specialists (classified into 1-2 years of experience and 3-4 years of experience).

**Table 3:** Disease or injury severity specific-least square mean estimates and standard errors by years of physician experience

	Board certified specialists	Non-board-certified specialists (1-2 years)	Non-board-certified specialists (3-4 years)	*p* for difference (Board certified specialists vs. 1-2 years)	*p* for difference (Board certified specialists vs. 3-4 years)
Mild, n	1208	462	368		
Time from arrival to assessment, min (SE)					
Crude	35.1 (1.02)	41.6 (1.03)	31.5 (1.04)	<0.001	0.02
Multivariate model**	38.0 (1.02)	44.0 (1.03)	33.6 (1.04)	<0.001	0.004
Time from assessment to point of change, min (SE)					
Crude	71.4 (1.02)	74.4 (1.03)	70.3 (1.03)	0.43	0.90
Multivariate model*	71.2 (1.01)	67.2(1.02)	73.6 (1.02)	0.01	0.26
Length of stay, min (SE)					
Crude	117.0 (1.01)	128.4 (1.02)	112.1 (1.03)	<0.001	0.24
Multivariate model**	125.7 (1.02)	135.2 (1.02)	118.6 (1.03)	0.008	0.07
Moderate, n	642	296	181		
Time from arrival to assessment, min (SE)					
Crude	35.4 (1.03)	35.0(1.05)	34.4 (1.07)	0.97	0.90
Multivariate model**	36.6 (1.03)	36.7 (1.05)	37.3 (1.06)	1.00	0.94
Time from assessment to point of change, min (SE)					
Crude	131.2 (1.02)	131.9 (1.03)	116.5 (1.04)	0.98	0.03
Multivariate model*	128.5 (1.02)	129.4(1.02)	121.4 (1.03)	0.97	0.19
Length of stay, min (SE)					
Crude	185.9 (1.02)	185.0 (1.02)	172.0 (1.03)	0.98	0.04
Multivariate model**	186.0 (1.02)	186.5 (1.02)	174.1 (1.03)	0.99	0.09
Severe, n	338	190	99		
Time from arrival to assessment, min (SE)					
Crude	26.2 (1.06)	33.3 (1.07)	30.9 (1.10)	0.02	0.26
Multivariate model**	33.0 (1.07)	41.0 (1.09)	36.4 (1.11)	0.01	0.55
Time from assessment to point of change, min (SE)					
Crude	85.7 (1.04)	92.2 (1.05)	71.7 (1.08)	0.46	0.06
Multivariate model*	81.5 (1.04)	80.3 (1.05)	72.4 (1.06)	0.94	0.10
Length of stay, min (SE)					
Crude	132.9 (1.03)	145.9 (1.03)	128.4 (1.05)	0.05	0.76
Multivariate model**	141.7 (1.04)	155.5 (1.04)	134.9 (1.05)	0.05	0.56

*Adjusted for patient's age, patient's sex, length of stay, and body region imaged. **Adjusted for patient's age, patient's sex, and body region imaged. Stratified the difference in severity of disease or injury in LOS by board certification status of the attending specialist.

**Table 4:** Body region imaged specific-least square mean estimates and standard errors by years of physician experience

	Board certified specialists	Non-board-certified specialist (1-2 years)	Non-board-certified specialists (3-4 years)	*p* for difference (Board certified specialists vs. 1-2 years)	*p* for difference (Board certified specialists vs. 3-4 years)
Head	1076	428	302		
Time from arrival to assessment, min (SE)					
Crude	27.7 (1.02)	30.8 (1.04)	25.4 (1.05)	0.04	0.18
Multivariate model**	24.9 (1.03)	28.7 (1.04)	22.8 (1.05)	0.003	0.15
Time from assessment to point of change, min (SE)					
Crude	83.9 (1.02)	86.6 (1.03)	76.5 (1.04)	0.66	0.08
Multivariate model*	87.5 (1.01)	82.5 (1.02)	87.0 (1.02)	0.01	0.96
Length of stay, min (SE)					
Crude	124.5 (1.02)	133.3 (1.02)	115.2 (1.03)	0.03	0.03
Multivariate model**	135.7 (1.02)	142.0 (1.02)	126.2 (1.03)	0.16	0.03
Chest	208	83	67		
Time from arrival to assessment, min (SE)					
Crude	26.7 (1.06)	24.7 (1.09)	27.8 (1.10)	0.69	0.91
Multivariate model**	25.6 (1.06)	24.9 (1.09)	27.2 (1.10)	0.94	0.81
Time from assessment to point of change, min (SE)					
Crude	99.6 (1.05)	104.8 (1.08)	105.3 (1.09)	0.80	0.80
Multivariate model*	100.3 (1.03)	104.1 (1.05)	101.0 (1.06)	0.76	0.99
Length of stay, min (SE)					
Crude	143.3 (1.03)	142.4 (1.05)	147.0 (1.06)	0.99	0.90
Multivariate model**	138.5 (1.03)	134.1 (1.05)	134.2 (1.05)	0.78	0.82
Abdominal	286	107	62		
Time from arrival to assessment, min (SE)					
Crude	40.1 (1.04)	49.3 (1.07)	43.9 (1.09)	0.02	0.57
Multivariate model**	45.1 (1.08)	55.5 (1.10)	49.1 (1.11)	0.01	0.60
Time from assessment to point of change, min (SE)					
Crude	84.3 (1.04)	92.2 (1.07)	65.5 (1.10)	0.46	0.03
Multivariate model*	81.1 (1.05)	78.8 (1.06)	71.0 (1.07)	0.82	0.07
Length of stay, min (SE)					
Crude	139.3 (1.03)	154.7 (1.05)	127.0 (1.06)	0.11	0.31
Multivariate model**	159.5 (1.05)	178.1 (1.06)	149.9 (1.07)	0.04	0.48
Full body	314	170	103		
Time from arrival to assessment, min (SE)					
Crude	50.9 (1.04)	52.0 (1.06)	46.6 (1.07)	0.93	0.46
Multivariate model**	51.0 (1.04)	52.7 (1.06)	47.2 (1.07)	0.85	0.54
Time from assessment to point of change, min (SE)					
Crude	99.3 (1.04)	102.6 (1.06)	86.4 (1.07)	0.86	0.16
Multivariate model*	94.8 (1.03)	95.9 (1.04)	93.6 (1.05)	0.97	0.97
Length of stay, min (SE)					
Crude	170.5 (1.02)	175.7 (1.03)	152.7 (1.04)	0.70	0.05
Multivariate model**	166.0 (1.02)	174.4 (1.03)	152.3 (1.04)	0.38	0.13
Other	304	160	114		
Time from arrival to assessment, min (SE)					
Crude	43.2 (1.04)	48.0 (1.06)	39.7 (1.07)	0.29	0.51
Multivariate model**	42.0 (1.05)	46.9 (1.06)	38.4 (1.08)	0.24	0.47
Time from assessment to point of change, min (SE)					
Crude	86.6 (1.04)	95.0 (1.05)	86.7 (1.06)	0.22	1.00
Multivariate model*	90.2 (1.02)	89.6(1.03)	93.5 (1.04)	0.98	0.65
Length of stay, min (SE)					
Crude	143.9 (1.03)	159.2 (1.04)	139.6 (1.05)	0.06	0.80
Multivariate model**	145.7 (1.03)	158.4 (1.04)	142.0 (1.05)	0.12	0.84

*Adjusted for patient's age, patient's sex, length of stay, and severity of disease *or injury*. **Adjusted for patient's age, patient's sex, and severity of disease *or injury*. Stratified the difference in body region imaged by CT in LOS by board certification status of the attending specialist.

## Discussion

This study is the first to report significant differences in time from arrival to assessment and LOS between patients treated by board-certified specialists and non-board-certified specialists, depending on the severity of their disease or injury and the region imaged by CT. For mild and severe cases managed by noncertified specialists with 1-2 years of experience who ordered head and abdominal CTs, the patients' time from arrival to assessment and total LOS were longer. In contrast, treatment by noncertified specialists with 3-4 years of experience was associated with a shorter LOS for mild cases than treatment by other categories of emergency physicians.

The longer LOS was probably associated mainly with a longer time from arrival to assessment. Noncertified specialists with 1-2 years of experience have less clinical experience and knowledge; for example, their ability to triage patients and determine injury or disease severity is usually limited. Therefore, they require more time to observe the patient and determine the need for CT. Additionally, severe cases require restoration of circulatory or respiratory dynamics. In these cases, the differences in time from arrival to assessment and LOS in the outpatient department were probably related to the physician's level of experience.

In abdominal cases, it is usually initially difficult to determine the need for testing because the symptoms are nonspecific. Furthermore, plain abdominal CT is an inferior test to contrast-enhanced CT, and it is difficult to determine whether contrast-enhanced CT is necessary^7, 9-15)^. Certified specialists perform abdominal CT more frequently than noncertified specialists, but they perform full-body CT less frequently. They were also associated with a shorter time between arrival and assessment of the patient and LOS than noncertified specialists with 1-2 years of experience. In other words, certified specialists tend to assess symptoms accurately and quickly and perform targeted imaging tests without delay. It is also possible that noncertified specialists routinely performed full-body CT scans on patients with abdominal symptoms without proper evaluation. Head CT has also been used as a routine test, which explain why patients attended by noncertified specialists with 3-4 years of experience had a shorter time from arrival to assessment than those attended by certified specialists.

Our study suggests the need to reduce the time from patient arrival to assessment to improve the quality of emergency healthcare. To reduce the LOS, support system triage training should be enhanced, a triage flow and decision score should be created^16-20)^, and the clinical examination skills of noncertified specialists should be improved^21)^. Previous studies have shown that appropriate triage flow and medical examination protocols^16-20)^ help to reduce LOS in emergency outpatient departments.

This study had some limitations. First, it was conducted in one medical facility only. A multi- institutional study would have been preferable. However, each hospital has different numbers of certified specialists and different support systems, making comparisons difficult. Second, cases of interest were not limited to referred emergency cases; therefore, complete elimination of selection bias was impossible. Third, since we did not classify referrals to other hospitals according to whether they involved injuries or not, we could not evaluate our findings according to the reason for referral. Fourth discrepancies in diagnoses by physicians and radiologists do occur, often in the Emergency Medicine Department^12, 22)^, delaying the clinical decision-making process regarding choice of treatment. But our study could not evaluate this influence. Finally, the presence of defective data was a limitation in this study. Indeed, this is a common problem in many observational studies. While there are statistical methods aimed at handling defective data, these methods are themselves based on a number of assumptions that were, in the case of the current study, challenging to investigate empirically.

## Conclusions

In the current system for attending patients in emergency departments, emergency physicians with minimal work experience are sometimes unable to work effectively, especially during the period from the arrival of a patient to their assessment. For physicians with minimal work experience, effective training using triage flow and medical examination protocols may help to reduce LOS in the emergency department.

## Funding

No funding was received.

## Author contributions

ST, TK, and SM designed and coordinated the study. ST analyzed data, and DU and AI provided technical assistance for the analysis. ST interpreted the data and drafted the manuscript. All authors read and critically revised manuscript and approved the final manuscript.

## Conflicts of interest statement

We declare that the research was conducted in the absence of any commercial or financial relationships that could be construed as a potential conflict of interest.

## References

[1] Zhang E, Hung SC, Wu CH, Chen LL, Tsai MT, Lee WH: Adverse event and error of unexpected life‐threatening events within 24hours of ED admission. Am J Emerg Med, 2017; 35: 479-483. 27974226 10.1016/j.ajem.2016.11.062

[2] Chang BP, Cain D, Mitroff SR: Emergency department crowding associated with differences in CXR interpretations between emergency physicians and radiologists. Am J Emerg Med, 2017; 35: 793-794. 28087098 10.1016/j.ajem.2016.12.067

[3] Lal NR, Murray UM, Eldevik OP, Desmond JS: Clinical consequences of misinterpretations of neuroradiologic CT scans by on-call radiology residents. AJNR Am J Neuroradiol, 21: 124-129. PMC7976358 10669236

[4] Soto JA, Anderson SW: Multidetector CT of blunt abdominal trauma. Radiology, 2012; 265: 678-693. 23175542 10.1148/radiol.12120354

[5] Broder J, Warshauer DM: Increasing utilization of computed tomography in the adult emergency department, 2000-2005. Emerg Radiol, 2006; 13: 25-30. 16900352 10.1007/s10140-006-0493-9

[6] Hautz WE, Kammer JE, Hautz SC, et al: Diagnostic error increases mortality and length of hospital stay in patients presenting through the emergency room. Scand J Trauma Resusc Emerg Med, 2019; 27: 54. 31068188 10.1186/s13049-019-0629-z PMC6505221

[7] Ilgen JS, Humbert AJ, Kuhn G, et al: Assessing Diagnostic Reasoning: A Consensus Statement Summarizing Theory, Practice, and Future Needs. Acad Emerg Med, 2012; 19: 1454-1461. 23279251 10.1111/acem.12034

[8] Siegel E, Groleau G, Reiner B, Stair T: Computerized follow-up of discrepancy in image interpretation between emergency and radiology department. J Digit Imaging, 1998; 11(3 Suppl 1): 18-20. 9735425 10.1007/BF03168250 PMC3453366

[9] Ding R, McCarthy ML, Desmond JS, Lee JS, Aronsky D, Zeger S: Characterizing Waiting Room Time, Treatment Time, and Boarding Time in the Emergency Department Using Quantile Regression. Acad Emerg Med, 2010; 17: 813-823. 20670318 10.1111/j.1553-2712.2010.00812.x

[10] Konnyu KJ, Kwok E, Skidmore B, Moher D: The effectiveness and safety of emergency department short stay units: a rapid review. Open Med, 2012; 6. PMC3329070 22567078

[11] Becker A, Segal G, Berlin Y, Hershko D: The emergency department length of stay; Is the time running out? Chin J Traumatol, 2019; 22: 125-128. 30956066 10.1016/j.cjtee.2019.01.008 PMC6543458

[12] Michael JT, Christopher MG, Joseph MD: Radiograph interpretation discrepancies in a community hospital emergency department. West J Emerg Med, 2019; 20: 626-632. 31316702 10.5811/westjem.2019.1.41375 PMC6625692

[13] Hastings RS, Powers RD: Abdominal pain in the ED: a 35 year retrospective. Am J Emerg Med, 2011; 29: 711-716. 20825873 10.1016/j.ajem.2010.01.045

[14] Powers RD, Guertler AT: Abdominal pain in the ED: Stability and change over 20 years. Am J Emerg Med, 1995; 13: 301-303. 7755822 10.1016/0735-6757(95)90204-X

[15] Modahl L, Digumarthy SR, Rhea JT, Conn AK, Saini S, Lee SI: Emergency Department Abdominal Computed Tomography for Nontraumatic Abdominal Pain: Optimizing Utilization. J Am Coll Radiol, 2006; 3: 860-866. 17412185 10.1016/j.jacr.2006.05.011

[16] Murrell KL, Offerman SR, Kauffman MB: Applying lean: implementation of a rapid triage and treatment system. West J Emerg Med, 2011; 12: 184-191. 21691524 PMC3099605

[17] Singh S, Awasthi S: Effect of In-Situation Versus Manchester Triage System-Based Initial Case Management on Hospital-Based Mortality: A Before and After Study. Indian J Pediatr, 2022; 89: 553-557. 35275337 10.1007/s12098-022-04092-5

[18] Weston V, Jain SK, Gottlieb M, et al: Effectiveness of Resident Physicians as Triage Liaison Providers in an Academic Emergency Department. West J Emerg Med, 2017; 18: 577-584. 28611876 10.5811/westjem.2017.1.33243 PMC5468061

[19] Burstrom L, Nordberg M, Ornung G, et al: Physician-led team triage based on lean principles may be superior for efficiency and quality? A comparison of three emergency departments with different triage models. Scand J Trauma Resusc Emerg Med, 2012; 20: 57. 22905993 10.1186/1757-7241-20-57 PMC3478190

[20] Ohana O, Soffer S, Zimlichman E, Klang E: Overuse of CT and MRI in paediatric emergency department. Br J Radiol, 2018; 91: 20170434. 29271231 10.1259/bjr.20170434 PMC6190788

[21] Xu T, Xu J, Yu X, Ma S, Wang Z: Clinical decision-making by the emergency department resident physicians for critically ill patients. Front Med, 2012; 6: 89-93. 22460453 10.1007/s11684-012-0183-9

[22] Mun JK, Min SS, Tae GS, et al: Evaluating the accuracy of emergency medicine resident interpretations of abdominal CTs in patinents with non-traumatic abdominal pain. J Korean Med Sci, 2012; 27: 1255-1260. 23091326 10.3346/jkms.2012.27.10.1255 PMC3468765

